# Youth Athlete Development and Nutrition

**DOI:** 10.1007/s40279-021-01534-6

**Published:** 2021-09-13

**Authors:** Ben Desbrow

**Affiliations:** School of Allied Health Sciences, Griffith University, Gold Coast, QLD Australia

## Abstract

Adolescence (ages 13–18 years) is a period of significant growth and physical development that includes changes in body composition, metabolic and hormonal fluctuations, maturation of organ systems, and establishment of nutrient deposits, which all may affect future health. In terms of nutrition, adolescence is also an important time in establishing an individual’s lifelong relationship with food, which is particularly important in terms of the connection between diet, exercise, and body image. The challenges of time management (e.g., school, training, work and social commitments) and periods of fluctuating emotions are also features of this period. In addition, an adolescent’s peers become increasingly powerful moderators of all behaviours, including eating. Adolescence is also a period of natural experimentation and this can extend to food choice. Adolescent experiences are not the same and individuals vary considerably in their behaviours. To ensure an adolescent athlete fulfils his/her potential, it is important that stakeholders involved in managing youth athletes emphasize eating patterns that align with and support sound physical, physiological and psychosocial development and are consistent with proven principles of sport nutrition.

## Key Points

**Table:** 

Advice to developing athletes regarding nutrition should prioritise long-term and sustainable health.
The pathway to elite sports performance is complex and is not dictated by undue emphasis on developing an individual’s body composition.

## Introduction

Regular exercise provides many benefits to adolescents, including social interaction, improved physical health, and the development of self-identity and self-esteem. In addition, the second decade of life is an important time in establishing an individual’s relationship with food and the lifelong connection between diet, exercise and body image [1]. This narrative review incorporates aspects of physiology, psychology, training science and sociology to describe our current understanding of the nutrition priorities for developing adolescent athletes. Given the pathway to elite adult performance is multifaceted [2] and non-linear (i.e., success at junior levels infrequently predicting elite adult performance [3]), sound nutrition supporting holistic athlete health is of utmost importance during this period. The responsibility for the provision of appropriate nutrition care to developing adolescent athletes is shared among sporting organisations, coaches, parents, teachers and the athletes themselves.

## The Changing Focus of Youth Athlete Development

Adolescence is a period of significant physical development that includes altered body composition, metabolic and hormonal fluctuations, maturation of organ systems and establishment of nutrient deposits, which may all affect future health [4]. Clearly, participation in sport plays an important role in supporting psychological well-being and developing a healthy self-image for most adolescents [5]. However, increased rates of disturbed eating attitudes/behaviours and body dissatisfaction have been evident in sports emphasizing leanness for many years [6–8]. While athletes may have intrinsic characteristics (e.g., perfectionism, high pain tolerance and motivation) valued by competitive sport, these qualities may also contribute to the development of disordered eating [9]. Furthermore, external pressures from coaches, peers, parents and social media also influence behaviour [10]. For example, previous findings suggest that careless comments from coaches referencing weight or appearance can precipitate the onset of, or perpetuate, disordered eating or eating disorders in athletes [11]. This evidence, combined with high-profile advocacy from former athletes [12] and a greater appreciation of the long-term health consequences of mismanaging the diet/exercise relationship in adolescent athletes [13], has led to recent calls to fundamentally change how sports manage aspiring athletes [14]. These changes may involve avoiding unwarranted nutrition/food-related discussions, abolishing body composition/weight assessments, raising awareness of the negative effects of chronic low energy availability (LEA), and disrupting toxic training environments featuring abusive body shaming, including the use of training strategies designed to manipulate an athlete’s physique independent of performance.

In response, some sporting organisations have attempted to safeguard their custodianship of adolescent athletes by publishing expected stakeholder behaviours. For example, in 2019, Gymnastics Australia released Body Positive Guidelines, providing specific recommendations on appropriate language, the frequency and delivery of nutrition education, and body composition assessment within gymnastic environments [15]. Specifically, the guidelines state that body composition assessments (including weight, height, skinfolds or physique assessments) should only be conducted by an experienced and certified anthropometrist, after education has been provided and written consent from gymnasts and parents/guardians has been obtained. The extent to which such strategies moderate the behaviour of coaches, parents and support staff, and/or ultimately reduce the incidence of undesirable health outcomes in developing athletes, is yet to be elucidated. In the interim, it seems prudent to suggest that those involved in adolescent sport require knowledge and support to ensure appropriate, evidence-based nutrition care is provided to developing athletes.

## Energy Needs of Developing Athletes

Throughout adolescence, adequate energy is required to meet both the growth and development needs of the individual, as well as the substrate demands associated with general physical activity, training and competition [16]. While group estimates of energy expenditure in adolescent athletes are available (i.e., males ~ 3640 ± 830, females ~ 3100 ± 720 kcal/day [17]), the energy expenditure of individual adolescent athletes may vary considerably. Changes in training and competition loads, participation in more than one competitive sport, part-time employment and/or concurrent compensatory sedentary behaviours may all impact energy needs. Determining the individual energy requirements of adolescent athletes is further complicated by metabolic and hormonal variability within and between individuals [18], as well as methodological difficulties in estimating both energy intake and energy expenditure [19].

Growth during puberty is directly related to the hormonal changes that accompany sexual development and is characterised by three phases: (1) minimal height velocity just before the spurt (prepubertal growth lag); (2) peak height velocity (PHV); and (3) decreasing height velocity (epiphyses fuse and final height is achieved) [20]. While girls generally start their growth spurt and attain PHV 2 years earlier than boys (~ 12 years for girls vs. ~ 14 years for boys), other factors such as ethnicity (e.g., individuals with European ancestry ~ 6 months younger skeletal age than chronologically matched individuals with Asian and African heritage [21]) may also influence the timing of growth.

The energy needs for growth (a component of the energy requirements of adolescent athletes) consist of two parts: the energy expended to synthesize new tissues, and the energy deposited in growing tissues [22]. The energy expended to synthesize new tissues can be directly measured via the doubly labelled water (DLW) method, or (more commonly) estimated indirectly via measures of resting metabolic rate (RMR). The use of adult-based equations to predict RMR in adolescent athletes is not recommended, as these have been shown to underestimate energy expenditure (up to 300 kcal/day) compared with indirect calorimetry measures [23]. Recently, new predictive resting metabolism equations have been developed from a cohort of male and female junior athletes (*n* = 126) who each undertook an indirect calorimetry assessment of RMR under standardised conditions. The cohort included athletes from a range of sports with an average age of 16.5 years (range 13.1–19.7) [24]. The predictive RMR equation for developing athletes was (Eq. 1):

$${\text{RMR }}\left( {{\text{kcal}}/{\text{day}}} \right) = {1}1.1 \; \times \;{\text{Body Mass }}\left( {{\text{kg}}} \right) + {8}.{4} \times {\text{Height }}\left( {{\text{cm}}} \right){-}\left( {{34}0{\text{ male or 537 female}}} \right).$$

The energy deposited in growing tissues is more difficult to measure but is considered small and is commonly estimated as ~ 2.0 kcal/g of daily weight gain (e.g. for a 15-year-old male gaining 6 kg/year =  ~ 33 kcal/day) [25]. Hence, while two energy components of growth may alter total caloric requirements, evidence suggests that changes associated with physical activity and/or athletic training are likely to have a much greater influence on total energy demands of adolescent athletes [22].

General energy requirements for adolescent populations with different levels of physical activity and/or training have been published [22]. At the individual level, energy expenditure can be accurately measured using methods such as DLW or via indirect calorimetry. Given these methods are expensive and rely on complex techniques, widely available methods for estimating individual energy expenditures warrant consideration. Published estimates of the specific energy cost of different exercises being undertaken by adolescent athletes do not currently exist. Consequently, the energy expenditure of exercise in adolescents is currently calculated by recording the type, intensity and duration of exercise, and, using the body weight of the individual, computing the energy cost using published adult values of metabolic equivalents (METs) for specific activities [26]. Wearable technologies incorporating accelerometers represent a relatively inexpensive alternative to estimate individual energy expenditure in younger populations. Recent reviews have summarised the validity of different ‘wearables’ to estimate total energy expenditure and the energy cost of physical activity against DLW [27] and indirect calorimetry [28] in younger (general) populations. These reviews suggest that (1) there is no current ideal device; (2) accelerometers tend to underestimate energy expenditure due to activities such as incline walking, bicycling and carrying items; and (3) more accurate results are recorded when the accelerometer is placed closer to the centre of mass (e.g., hip compared with wrist or ankle) of the individual.

The accurate determination of energy intakes and expenditures is important, as it appears that LEA and potential symptoms of relative energy deficiency in sport (RED-S) in young individuals undertaking heavy training is common [29, 30]. In developing athletes, LEA may lead to a number of serious health consequences, including delayed puberty, menstrual irregularities, poor bone health, short stature, the development of disordered eating behaviours, and increased risk of injury [31]. Furthermore, in females with a gynecological age of ≤ 14 years, the effects of LEA may be more pronounced [32]. Conversely, some developing athletes (e.g. in throwing events) demonstrate anthropometric characteristics consistent with chronic disease risk [33]. In this context, severe and prolonged energy restriction is not recommended, with weight maintenance, rather than weight loss, considered a more appropriate management strategy in developing individuals [1].

## Macronutrient Needs of Developing Athletes

### Protein

Adolescents require protein to support general growth and development [16], in addition to enhancing the response to exercise training [34]. During peak growth, increases in lean body mass can reach ~ 2.3 g/day in females and ~ 3.8 g/day in males, which represents an approximately threefold increase from the prepubertal period [35]. In addition, longitudinal data indicate that physically active youth accrue greater increases in lean body mass than their sedentary peers [36]. While regular training does not appear to influence protein turnover in early adolescence [37], one proposed explanation for the enhanced lean mass deposition observed during puberty relates to enhanced anabolic sensitivity (i.e., a greater efficiency of dietary protein utilisation) [38]. This theory has received further support by the recent demonstration that adolescents (males and females) had a greater whole-body net balance when provided with small to optimal amounts of post-exercise protein than weight-stable adults [39]. Furthermore, an increased efficiency of amino acid use would explain previous nitrogen-balance studies that did not demonstrate additional dietary protein intakes were required in adolescent sprint athletes to maintain a positive nitrogen balance during their peak growth phase [40]. Total energy intake is an important consideration in the assessment of protein requirements. With suboptimal energy intake, endogenous protein is mobilised, as well as liver glycogen, to maintain homeostasis of blood glucose, potentially reducing the availability of protein for its primary functions [41]. Provided adequate energy is being consumed, it appears that protein recommendations to maximise whole-body net balance after exercise are primarily influenced by total body and fat-free mass. Protein intake at ~ 0.11 g/kg/h during post-exercise recovery, or the equivalent of ~ 1.5 g/kg/day (e.g. ~ 0.3 g protein/kg × 5 meal times), should be sufficient to replace any exercise-induced amino acid oxidative losses, enhance whole body net protein balance, and support the normal growth and development of adolescent athletes [39, 40].

### Carbohydrate

The duration and intensity of exercise sessions determines carbohydrate (CHO) utilisation patterns and refuelling requirements [42]. In addition, the availability of exogenous and endogenous CHO influences exercise-mediated training adaptations [43]. Existing evidence suggests that the utilisation of CHO in adolescents does not differ substantially from that of adults (for review see Desbrow and Leveritt [44]). While the impact of developmental age on CHO-mediated training adaptations remains unclear, a reduced capacity to adapt to changes in CHO availability observed in obese versus non-obese adults [45] also appears evident in those aged 8–17 years [46]. The mechanisms underpinning the ergogenic effect of CHO in adults are metabolic (i.e., substrate provision), and centrally derived (i.e., CHO signalling in the oral cavity) [47]. To date, no study has assessed the impact of centrally mediated CHO effects in adolescent athletes.

Dietary CHO needs should be considered in light of the training loads and competition characteristics that are typically undertaken by adolescent athletes. These can differ from those undertaken by adult athletes in a number of ways. First, adolescent athletes may be involved with numerous organisations (e.g., schools, clubs and regions) creating different competition frequencies and formats, such as sports carnivals, representative events and trials. It is also common for aspiring adolescent athletes to participate in a number of different sports. These different energy demands and subsequent CHO requirements must be considered, particularly when the participation in different sports is concurrent.

During short bouts of exercise (i.e. < 1 h in duration), a CHO mouth rinse or small amounts of CHO may benefit performance, particularly if the athlete has not eaten prior to the exercise. During more prolonged physical activity, a single CHO source (i.e., glucose) can be oxidised at rates up to ~ 0.6 g/kg/h in young boys, suggesting a 12-year-old male with a bodyweight of 45 kg could utilise ⁓30 g of CHO/h from dietary sources. While dietary strategies incorporating the use of refined CHO to manipulate metabolism during endurance sport (e.g., CHO loading and/or multiple transportable forms of CHO throughout exercise) are likely to improve performance in events of > 90 min duration [48], these recommendations should only be employed in relevant situations, which are less likely for adolescent athletes given the shorter duration of many events.

### Fat

Adequate dietary fat intake is needed to meet the requirements for fat-soluble vitamins and essential fatty acids, and helps to provide energy to support growth and maturation [18]. In addition, evidence suggests that maximal fat oxidation rates (relative to lean mass) appear slightly higher in athletes < 18 years [49]. To date, dietary strategies promoting the role of the intramuscular triacylglycerols on performance and the effect of training in a CHO-depleted state on adolescent endurance athletes remains unstudied. Since chronically high fat intakes are associated with increased chronic disease risk, the recommendation for type and total fat intake by adolescent athletes remains in accordance with public health guidelines. Typically, these guidelines suggest a dietary fat intake of 20–35% of total energy, with saturated trans fatty acids providing no more than 10% of total energy intake [50, 51].

When energy demands change, sports nutrition recommendations encourage athletes to manipulate dietary intake to support daily performance and optimise adaptations to training [52], a concept known more commonly as ‘fuel for the work required’ [53]. Adolescent athletes are likely to require support to develop a ‘food first’ approach to matching energy intakes with increased training loads and may benefit from practical resources translating changes in macronutrient needs to food selection [54].

## Micronutrient Needs for Adolescent Athletes

### Iron

Depleted iron stores, without clinical symptoms, are observed frequently in studies conducted on adolescent athletes (particularly endurance competitors) [55, 56]. However, interpreting one-off measures of iron status markers (e.g., serum ferritin) in developing athletes should be done with caution for several reasons: cut-off values for ferritin are not standardised in studies of youth athletes; athletes generally have lower levels of ferritin than non-athletes; sex differences are evident between males and females during adolescence; and ferritin levels can be falsely positive in mild infection, injury or physiological stress [57].

Detection and early treatment of iron depletion in adolescent athletes is nonetheless warranted. This is because growth increases iron requirements in adolescents compared with older athletes, resulting in the progression from low iron stores to a state of iron deficiency being rapid. Reference values (Table 1) [58] and strategies to address poor iron status in athletes [59] have been established.

**Table 1:** Definition and assessment of stages of iron deficiency [58]

Stage	1. Depleted iron stores	2. Early functional iron deficiency	3. Iron deficiency anaemia
Description	Iron stores in the bone marrow, liver, and spleen are depleted	Erythropoiesis diminishes as the iron supply to the erythroid marrow is reduced	Haemoglobin production falls, resulting in anaemia
Assessment	SF < 35 µg/L Hb > 115 g/L TS > 16%	SF < 20 µg/L Hb > 115 g/L TS < 16%	SF < 12 µg/L Hb < 115 g/L TS < 16%

*SF* serum ferritin, *Hb* haemoglobin, *TS* transferrin saturation

In terms of performance, even mild tissue decrements in iron have the potential to adversely affect endurance capacity and aerobic adaptation to training [60]. Indeed, a recent study involving over 70 adolescent female athletes indicated a moderate relationship between athletic performance, the concentration of soluble transferrin receptors, and dietary iron intake, emphasizing the importance of iron intake for aspiring adolescent female athletes [61].

In adolescent female endurance athletes, suboptimal iron status is mainly attributed to low iron intake and low iron bioavailability in combination with high requirements associated with training and blood loss (e.g., increased red cell mass, menstruation, haematuria, haemolysis) [62, 63]. In contrast, suboptimal iron status in adolescent male athletes is associated more with high physiological requirements (i.e., training and growth) than with diet. Recommendations for iron for developing girls account for iron lost from menstruation. While often a population reference value is used as an age cut-off for menarche (e.g. 14 years), individual recommendations should be adjusted when individual differences exist (particularly when menarche occurs earlier). It is possible that vegetarian athletes have increased requirements due to low iron bioavailability of non-haem iron sources. However, scientific advisory summaries indicate that when comparing vegetarians with non-vegetarians, most studies demonstrate no significant differences in dietary iron intake or haemoglobin concentrations. Although serum ferritin concentrations are consistently statistically significantly lower in vegetarians, they are usually within the reference ranges [64]. That said, it seems prudent to ensure adolescent vegetarian athletes monitor iron status routinely.

### Calcium

Lifespan calcium requirements are highest during the pubertal growth spurt. The rate of skeletal calcium accretion during adolescence is estimated to be around 300 mg/day [65]. There are currently no specific recommendations for calcium intake for athletes, therefore, until further studies are undertaken, population reference standards can be used as a benchmark for assessing adequacy. Calcium recommendations are based on estimates of urinary and sweat losses and assume a net calcium absorption from food (often ~ 25–35%). Recommendations for adolescents vary between regions, with values ranging from 800 mg/day (e.g., UK females aged 15–18 years) to 1300 mg/day (US, Canada, Australia for males and females aged 14–18 years). High-intensity weight-bearing exercise and, to some extent, resistance exercise, increase bone mineral content in exercising adolescents. [66–68]. While this effect is small (typically < 6% difference) and unlikely to increase calcium requirements, enhanced bone mineral content may maximise peak hip strength and prevent osteoporosis in later life.

### Vitamin D

While vitamin D is best known for its role in bone health, it has many functions in other physiological systems (e.g., immune system, muscular system). Vitamin D insufficiency is also linked to skeletal muscle function, muscle pain and weakness, and inflammation, and may potentially increase susceptibility to injury and slow rate of rehabilitation from injury (for review see de la Puente Yague et al. [69]). Hence, vitamin D status (particularly in adult athletes) has received considerable recent scientific attention. Currently, the influence of vitamin D status and the benefits of supplementation in youth athletes identified as deficient remain largely unknown. However, recent prospective studies suggest little correlation between serum levels of 25 hydroxyvitamin D and sports performance in adolescent athletes [70], even when vitamin D deficiencies are corrected [71].

Similar to adult athletes, developing athletes are at high risk of vitamin D deficiency if they have experienced limited sun exposure (e.g., reside in latitudes > 35 °, spend long periods training indoors, have dark skin, use sunscreen or wear protective clothing). Typically, fixed amounts of vitamin D are recommended beyond infancy, until values for older adults are further increased to account for the reduced capacity of the skin to produce vitamin D with ageing. Recommendations for vitamin D differ by region (Australia = 5 μg/day, US/Canada = 15 μg/day, European countries range from 10 to 20 μg/day). However, all authorities agree that monitoring of 25 hydroxyvitamin D is important for at-risk groups.

## Fluid Needs of Developing Athletes

Young individuals appear to have similar capacity to adults to deal with thermal loads and exercise tolerance time during exercise in the heat [72, 73]; however, the mechanisms by which young individuals dissipate heat loads during exercise differ from those of adults [73, 74]. Children and adolescents appear to rely more on peripheral blood redistribution (radiative and conductive cooling) rather than sweating (evaporative cooling) to maintain thermal equilibrium [74, 75]. There is also evidence that adolescents who undertake regular training adapt by enhanced peripheral vasodilation [76], which is likely to improve non-evaporative cooling. While the timing of the transition from child-like to adult-like thermoregulatory mechanism is likely to be related to pubertal development, it appears that these changes do not become physiologically evident until puberty has been completed [77].

There is some evidence suggesting an increased prevalence of heat illness associated with sport and activity in youth athletes [78]. Heat illness may be influenced by poor hydration status along with other factors such as undue physical exertion, insufficient cooling between exercise bouts and inappropriate choices of clothing, including uniforms. Unfortunately, there is no evidence to determine the extent to which (if at all) fluid intake may modulate the risk of heat illness in adolescent athletes. This is because fluid monitoring studies on children and adolescents at risk of heat illness are scarce and often fail to report participants who actually experience heat illnesses [79]. In contrast, field studies [80] and large cohort investigations [75] indicate that trained adolescent athletes can experience significant deficits in fluid (> 4% body weight) and high sweat rates (≤ 2.16 L/h), respectively, in response to exercise. Fluid shifts of this magnitude have the potential to induce signs/symptoms of hypohydration and affect exercise performance. Consequently, fluid intake guidelines for young athletes [81, 82] are similar to those recommended for adults [83]. This advice includes commencing exercise well-hydrated, developing individualised drinking plans (refined regularly during puberty to accommodate changes in sweat rate), limiting body mass losses during activity to ≤ 2% from pre-exercise values and avoiding weight gain. In general, fluid intakes of 13 mL/kg per hour of exercise should be sufficient to avoid significant fluid deficits in developing athletes [82].

Finally, developing athletes may not recognise the signs or symptoms of heat stress, forget to drink unless reminded and continue to exercise to keep up with their peers. Therefore, it is important that when environmental and contextual factors (e.g., intensity/duration of exercise, clothing, fluid availability) combine to increase thermoregulatory risks, strategies to moderate metabolic heat loads in adolescent athletes should be implemented proactively.

## Dietary Supplements and Ergogenic Aid Use by Developing Athletes

The judicious use of specific dietary supplements and nutritional ergogenic aids may improve sporting performance in adults [84]. However, most (but not all [85]) products are yet to have their effectiveness and long-term safety rigorously explored in younger populations, often due to the ethical principle of beneficence (i.e. cost vs. benefit). Despite the paucity of scientific evidence, reported supplement use with the intent to improve sports performance among youth athletes is common [86, 87]. The prevalence of supplement use among US children and adolescents (< 18 years) as a performance enhancer was 1.6% [86]. Vitamin/mineral supplements, sports/protein powders, vitamin waters, creatine and caffeine are commonly identified as popular supplements used by adolescent athletes [88].

Adolescent athletes take ‘performance-enhancing’ dietary supplements for several reasons, such as pressure to achieve results, the pursuit of physical ideals, and peer, social and marketing pressure. Furthermore, while some elite adolescent athletes do not believe that dietary supplements are required to be successful in their sport, they still consider them important for certain training adaptations, such as strength gains [89].

Generally, it is considered inappropriate for young athletes to be encouraged to consume dietary supplements for performance enhancement. This view is consistent with those of leading sporting organisations and expert groups [90–92]. This recommendation excludes the clinical use of dietary supplements (e.g., calcium, iron, vitamin D) when taken under appropriate guidance from suitably qualified health professionals (e.g., a medical practitioner, sports dietitian). Developing athletes have the potential for large performance gains through maturation and experience in their sport, along with adherence to proper training, nutrition and rest regimens. Apart from issues related to safety, the use of legal supplements in developing athletes over-emphasizes their ability to manipulate performance (i.e., typically 2–5% improvement [84]). Prioritising prudent training and whole food-based nutrition practices in adolescent athletes has been emphasized, even when safe supplements are being considered [85].

## Conclusion

Adolescent athletes have unique nutritional requirements as a consequence of undertaking daily training and competition in addition to the demands of growth and development. Dietary education and recommendations for this group of athletes should reinforce eating for long-term health. More specifically, the developing athlete should be encouraged to moderate eating patterns to reflect daily exercise demands and provide a regular spread of high-quality CHO and protein sources over the day, especially in the period immediately after training. Particular consideration should also be given to the potential risk of LEA and also to dietary calcium, vitamin D and iron intake of youth athletes because of the risk of deficiency and high requirements. The nutrient needs of adolescent athletes should be met by food rather than supplements. The use of dietary supplements by developing athletes over-emphasizes their ability to manipulate performance in comparison with other training and dietary strategies.
